# Genetic analysis and prenatal diagnosis in a Chinese with growth retardation, abnormal liver function, and microcephaly

**DOI:** 10.1002/mgg3.1751

**Published:** 2021-07-31

**Authors:** Peiwei Zhao, Lei Zhang, Li Tan, Sukun Luo, Yufeng Huang, Hanming Peng, Jiangxia Cao, Xuelian He

**Affiliations:** ^1^ Precision Medical Laboratory Wuhan Children's Hospital (Wuhan Maternal and Child Healthcare Hospital) Tongji Medical College Huazhong University of Science & Technology Wuhan PR China; ^2^ Gastroenterology Department Wuhan Children's Hospital (Wuhan Maternal and Child Healthcare Hospital) Tongji Medical College Huazhong University of Science & Technology Wuhan PR China; ^3^ Prenatal Diagnosis Center Wuhan Children's Hospital (Wuhan Maternal and Child Healthcare Hospital) Tongji Medical College Huazhong University of Science & Technology Wuhan PR China

**Keywords:** abnormal liver function, COG6, congenital disorders of glycosylation, hypohidrosis, microcephaly

## Abstract

**Background:**

Congenital disorders of glycosylation (CDG) are a genetically heterogeneous group of disorders caused by defects in the synthesis and processing of glycoproteins. COG6‐CDG is a kind of disorder caused by conserved oligomeric golgi complex 6 (COG6) deficiency. To date, only 19 patients with COG6‐CDG have been reported.

**Methods:**

We report a girl in a Chinese family with developmental delay, growth retardation, microcephaly, abnormal liver function, and hypohidrosis. Trio whole‐exome sequencing was performed for this patient and her parents, and the variants identified were validated by Sanger sequencing. Prenatal diagnosis was done for this family during a subsequent pregnancy. The literature review on these patients was performed by reviewing articles published in English and Chinese.

**Results:**

Genetic sequencing identified two novel heterozygous mutations: c.428G>T (p.S143I) and c.1843C>T (p.Q615X) in the *COG6* gene, inherited from her healthy parents, respectively. A total of 11 different mutations in *COG6* have been reported previously, and mutations potentially affecting splicing are the most common. The main clinical features included development delay, facial dysmorphism, growth retardation, skin abnormalities (hypohidrosis), microcephaly, abnormal brain structure, liver involvement, and recurrent infections.

**Conclusion:**

Our work broadens the mutation spectrum of *COG6* gene and states the importance of whole‐exome sequencing in facilitating the definitive diagnosis of this disorder and prenatal diagnosis in a subsequent pregnancy.

## Graphical abstract

We report a patient from China with growth retardation, abnormal liver function, hypohidrosis, and microcephaly. Genetic sequencing identified two novel heterozygous mutations: c.428G>T (p.S143I) and c.1843C>T (p.Q615X) in the COG6 gene, inherited from the parents. To date, only 19 patients with COG6‐CDG have been reported.

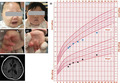

**Abbreviations**
ACMG: American College of Medical Genetics and Genomics
CDGs: congenital disorders of glycosylation
COG6: conserved oligomeric golgi complex 6
ECG: echocardiography
EEG: electroencephalogram
PCR: polymerase chain reaction

## 1 INTRODUCTION

Congenital disorders of glycosylation (CDGs) are a large and very heterogeneous group of metabolic disorders that comprise defects in glycosylation of proteins or lipids (Van Scherpenzeel et al., 2016). Glycosylation is the principal form of posttranslational modification of proteins and lipids, and it plays an important role in protein folding, molecular recognition, and other physiological processes (Bobby et al., 2018; Freeze et al., 2012). Because glycosylation occurs in every cell in every organism, CDGs is a multisystem disease that usually involves the nervous system, liver, heart, eyes, skeleton, and immune system (Alsubhi et al., 2017; Leroy, 2006; Li et al., 2019). The clinical phenotypes of CDG range from mild symptoms to severe multisystem dysfunction and even death (Jaeken, 2011).

Conserved oligomeric golgi (COG) complex plays an essential role in the maintenance of the structure of golgi apparatus, trafficking, and glycosylation of proteins. The COG complex is a heterooctamer containing eight different subunits (COG1–COG8) that are organized into lobe A (from COG1 to COG4) and lobe B (from COG5 to COG8) (Ungar et al., 2005). Since 2004, except for COG3, deficiency in other seven subunits have been reported to be associated with CDG (Barone et al., 2014; Haijes et al., 2018; Li et al., 2019; Lübbehusen et al., 2010; Rymen & Jaeken, 2014; Ungar et al., 2005; Wang et al., 2020).

COG6‐CDG (MIM: 614576) is a rare CDG type, since Lubbehusen and colleagues described the first COG6‐CDG in 2010 (Lübbehusen et al., 2010), to our knowledge, 19 cases have been reported (Alsubhi et al., 2017; Huybrechts et al., 2012; Li et al., 2019; Lübbehusen et al., 2010; Shaheen et al., 2013; Wu et al., 2017). In this study, we describe a Chinese girl with developmental delay, microcephaly, and abnormal liver function carrying two novel compound‐heterozygous mutations in *COG6* gene (OMIM 606977) inherited from her parents, respectively. Prenatal diagnosis was performed for this family during subsequent pregnancy and revealed the fetus is carrying one mutation from her father. In addition, we summarized the clinical manifestations of patients with COG6‐CDG by reviewing articles published in English and Chinese.

## 2 CLINICAL REPORT

The proband was admitted to the Rehabilitation Department in Wuhan Children's Hospital due to psychomotor delay at 8 months old. She was the only child of healthy nonconsanguineous parents without relevant family medical history. A small biparietal diameter in utero was detected by ultrasound examination during the last trimester of pregnancy. She was born at 39 weeks of gestation by spontaneous vaginal delivery with a birth weight of 3.25 kg, birth length of 48 cm. Head circumference (HC) and Apgar scores were not available. At the age of 1 month, her brain MRI did not reveal any obvious abnormality.

**FIGURE 1:**
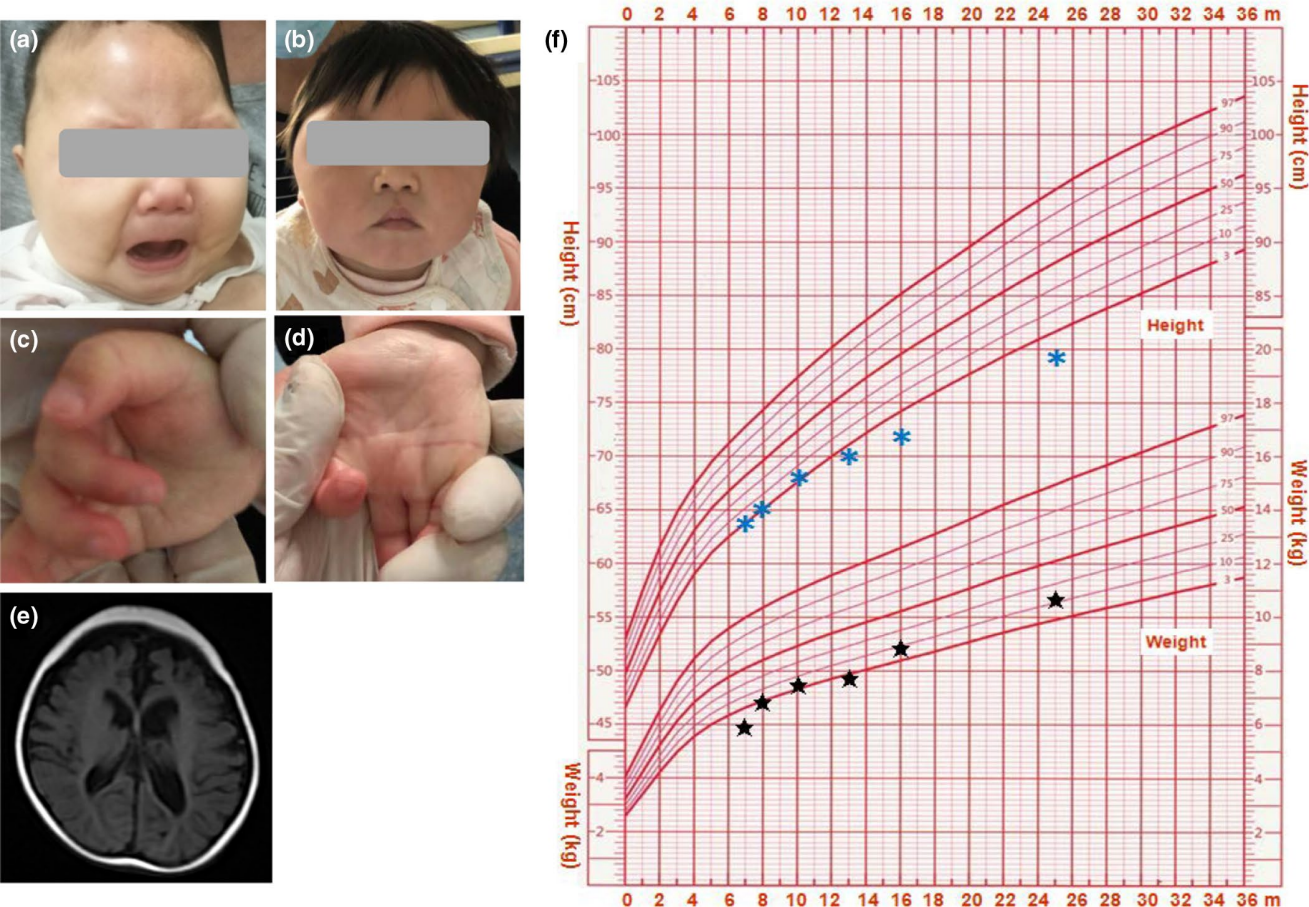
Abnormal facial appearance at the age of 8 months (a) and 20 months (b); (c,d) Adducted thumbs and camptodactyly. (e) Lesions of bilateral temporal, occipital, and parietal lobes and enlarged cerebral fissure and lateral ventricles. (f) Growth curves of this patient; the blue asterisks indicate height and the black stars indicate weight

On admission, she was noticed to have growth retardation, with a height of 65 cm (3rd–10th centile), and a weight of 7 kg (3rd–10th centile), and microcephaly with an HC of 36.4 cm (3rd centile). She had obvious dysmorphic features, including a narrow forehead, hypertelorism, bulbous nose, and downslanting palpebral fissures (Figure 1a,b). She had a severe psychomotor delay, hypotonia, and was unable to control her head, turn over, and sitting steadily. Her hands were clinched and both thumbs were adducted (Figure 1c,d). She had a response to sound and her eyes could follow the light. Her skin was dry and was unable to sweat. Her liver and spleen were impalpable.

She was extensively investigated by blood biochemistry examinations, echocardiography, electroencephalogram, brain MRI, and growth and development scale (Gesell Developmental Schedules). Blood biochemistry examinations showed abnormal liver function with elevated total billirubin (41.4 μmol/L, reference: 2–19 μmol/L), glutamic oxalacetic transaminase (231 U/L, reference: 10–40 U/L), glutamic pyruvic transaminase (72 U/L, reference: 7–45 U/L), and alkaline phosphatase (399 U/L, reference: 42–220 U/L). Renal function test showed slightly decreased blood creatinine (15.1 μmol/L, reference: 44.2–132.6 μmol/L) and urea nitrogen (1.76 mmol/L, reference: 2.9–7.1 mmol/L). The other examinations, including immune, thyroid function, coagulation, and metabolism screening test for amino acid in blood and organic acid in the urine, were normal. Echocardiography (ECG) revealed a mild atrioventricular block, and electroencephalogram (EEG) showed localized sharp waves and slow spindle waves during sleep, but no seizure. An electrocardiogram showed a shunt from left to right, indicating the likelihood of patent foramen ovale or atrial septal defect and. Brain MRI displayed softening lesion of bilateral temporal, occipital, and parietal lobes and enlarged cerebral fissure and lateral ventricles (Figure 1e). Gesell's developmental scale indicated that the patient was delayed in language, motor development, and social interaction.

She had a history of fever and abnormal liver function with a higher glutamic‐pyruvic transaminase (136 U/L) and glutamic oxalacetic transaminase (509 U/L) at the age of 7 months old, had been hospitalized in Gastrointestinal Department, and received treatment with inosine and atomolan. She also had recurrent upper respiratory tract infections, and gastrointestinal dysfunction, such as chronic diarrhea. At the age of 2 years old, her growth retardation was obvious, with a height of 79 cm (<3rd centile), a weight of 11 kg (3rd–10th centile; Figure 1f), and an HC of 41.5 cm (<3rd centile), and she was still unable to sit and walk independently.

## 3 METHODS

### 3.1 Trio whole‐exome sequencing and Sanger sequencing verification of the *COG6* genetic mutation

After obtaining written informed consent, genomic DNAs of this patient and her parents were extracted from whole blood samples using the QIAamp Blood DNA mini kit (Qiagen) according to the manufacturer's instructions. Whole‐exome sequencing and subsequent data analysis were conducted with the help of the third‐party medical testing laboratory (Chigene Lab). Candidate variants in the *COG6* gene were verified by Sanger sequencing using self‐designed primers. The primers for amplification of the *COG6* gene (NG_028352.1) were synthesized by Sangon Biotechnology Co. Ltd. The primer sequences for exon 4 were forward 5′‐GAG TTC CAT AGA GTG ATC TC‐3′ and reverse 5′‐CAT CAT TTC TGA ACT CCA CAG C‐3′, and the primer sequences for exon 19 were forward 5′‐GAT TAA CTG TGT AGC CAT ATA GTG‐3′ and reverse 5′‐GGA TTC ATC ACG GCT GCA TAC‐3′. The target fragments were amplified using polymerase chain reaction (PCR) and the products were sequenced using an ABI 3500DX sequencer (Applied Biosystems). Prenatal diagnosis for this family was performed by amniocentesis in the subsequent pregnancy.

## 4 RESULTS

### 4.1 Whole‐exome sequencing and prenatal diagnosis

**FIGURE 2:**
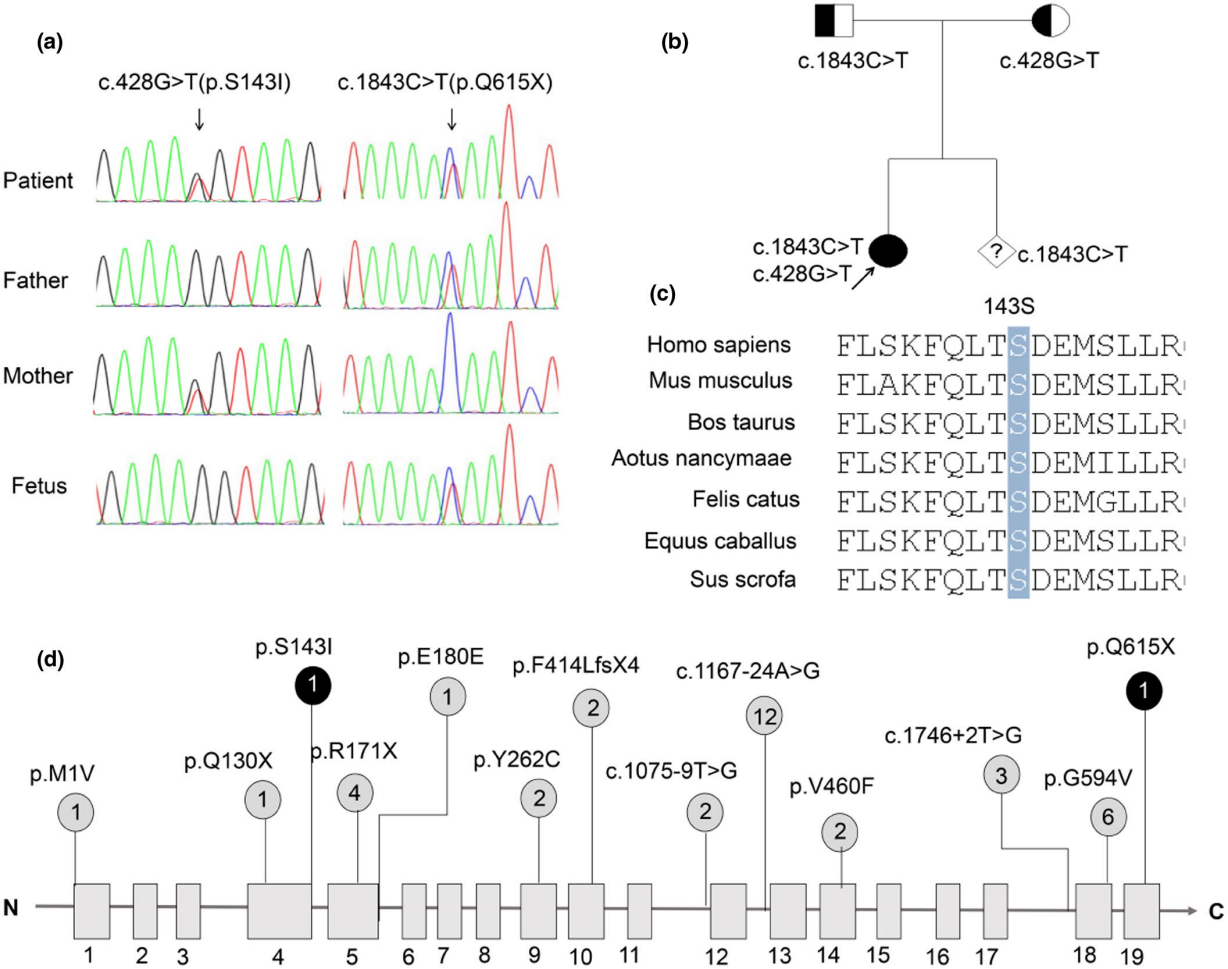
(a) Sanger sequencing of *COG6* mutations in this family, (b) genealogical tree of this family, (c) conservation analysis of COG6 protein among different species. The 143rd amino acid is indicated by a blue bar and conserved throughout all indicated species. (d) Distribution of variants within the *COG6* gene. Variants described previously are in gray, and variants in our study are in black. The numbers in circle indicate that the total alleles of each variant reported in COG‐CDG patients

Whole‐exome sequencing detection and subsequent data analysis found two heterozygous mutations: c.428G>T (p.S143I) in exon 4 and c.1843C>T (p.Q615X) in exon 19 of *COG6* (NM_020751.3) in the patient, inheriting from his mother and his father (Figure 2a,b), respectively, which was confirmed by Sanger sequencing. Two mutations are not listed in any database (i.e., gnomAD, ClinVar, and 1000 genomes). Bioinformatic analysis showed these two residues are conserved among different species (Figure 2c). According to the standards and guidelines recommended by the American College of Medical Genetics and Genomics (ACMG) (Richards et al., 2015), these two mutations were classified as likely pathogenic and pathogenic, respectively.

After 1 year, the mother was pregnant again, and ultrasonographic screening during the subsequent pregnancy did not reveal any abnormal findings. The gene testing using amniotic fluid cells at 16 weeks of pregnancy showed that the fetus only carried the c.1843C>T mutation (Figure 2a). and continued the pregnancy. Following up, the child was born and no abnormality is found at present.

## 5 DISCUSSION

Glycosylation is an essential biological process in cells for various protein or lipid modifications and ~2% of human genes encode glycosylation‐related proteins (Apweiler et al., 1999). CDGs affect many organs and it is difficult to make a definitive diagnosis because of lack of uniformity in clinical manifestations. In this study, we reported a Chinese patient with growth retardation, facial dysmorphism, microcephaly, developmental delay, abnormal liver function, hypohidrosis, recurrent infection, and gastrointestinal dysfunction. Two variants in *COG6* gene were found in this patient, and bioinformatic analysis suggested that these two variants are likely pathogenic and pathogenic, respectively. One is nonsense mutation, c.1843C>T (p.Q615X), which gives rise to premature termination of translation and truncated protein, whereas missense mutation, c.428G>T (p.S143I), may result in misfolding and degradation of the mutated protein. Another *COG6* missense mutation, c.1646G>T(p.G594V), was proved to lead to decreased protein due to instability of the *COG6* mRNA (Lübbehusen et al., 2010). The missense mutation, c.428G>T (p.S143I), in our patient, caused polar amino acids to be replaced by hydrophobic amino acids, nonpolar amino acids, which could affect the structure or stability of protein, consequently, disrupt the function of COG6 protein.

As shown in Figure 2d, to date, a total of 13 different variants have been reported in 20 COG6‐CDG patients from Saudi (9), Morocco (4), Turkey (3), Bulgaria (1), and China (3), respectively. Of these variants, 3 are splicing variants, 3 are nonsense mutations, and 5 are missense mutations, 1 is an insertion of one base which results in a frameshift and immediate stop codon, and 1 is synonymous mutation (Alsubhi et al., 2017; Huybrechts et al., 2012; Li et al., 2019; Lübbehusen et al., 2010; Wu et al., 2017). The splicing variant c.1167‐24A>G, which results in a frameshift and insertion of the premature stop codon (NM_020751.2: p.G390Ffs*6), was the most common and found to be homozygous in six patients with COG‐CDG (Alsubhi et al., 2017; Shaheen et al., 2013), which qualifies c.1167‐24A>G as a hotspot variant in the *COG6* gene. Interestingly, of all 20 patients with COG‐CDG, 13 are homozygous carriers for COG6 mutations (Alsubhi et al., 2017; Huybrechts et al., 2012; Shaheen et al., 2013; Ungar et al., 2005), and 10 out of 13 are from Middle East, thus, intraethnic marriage may be an important factor for the relative prevalence of this disorder in these regions. It should be noted that one patient was the sister of the patient (Lübbehusen et al., 2010). She did not have a genetic test and died at the age of 5 weeks. According to her clinical manifestations, this patient was suspected to carry the same mutation (c.1646G>T) as her elder sister.

**FIGURE 3:**
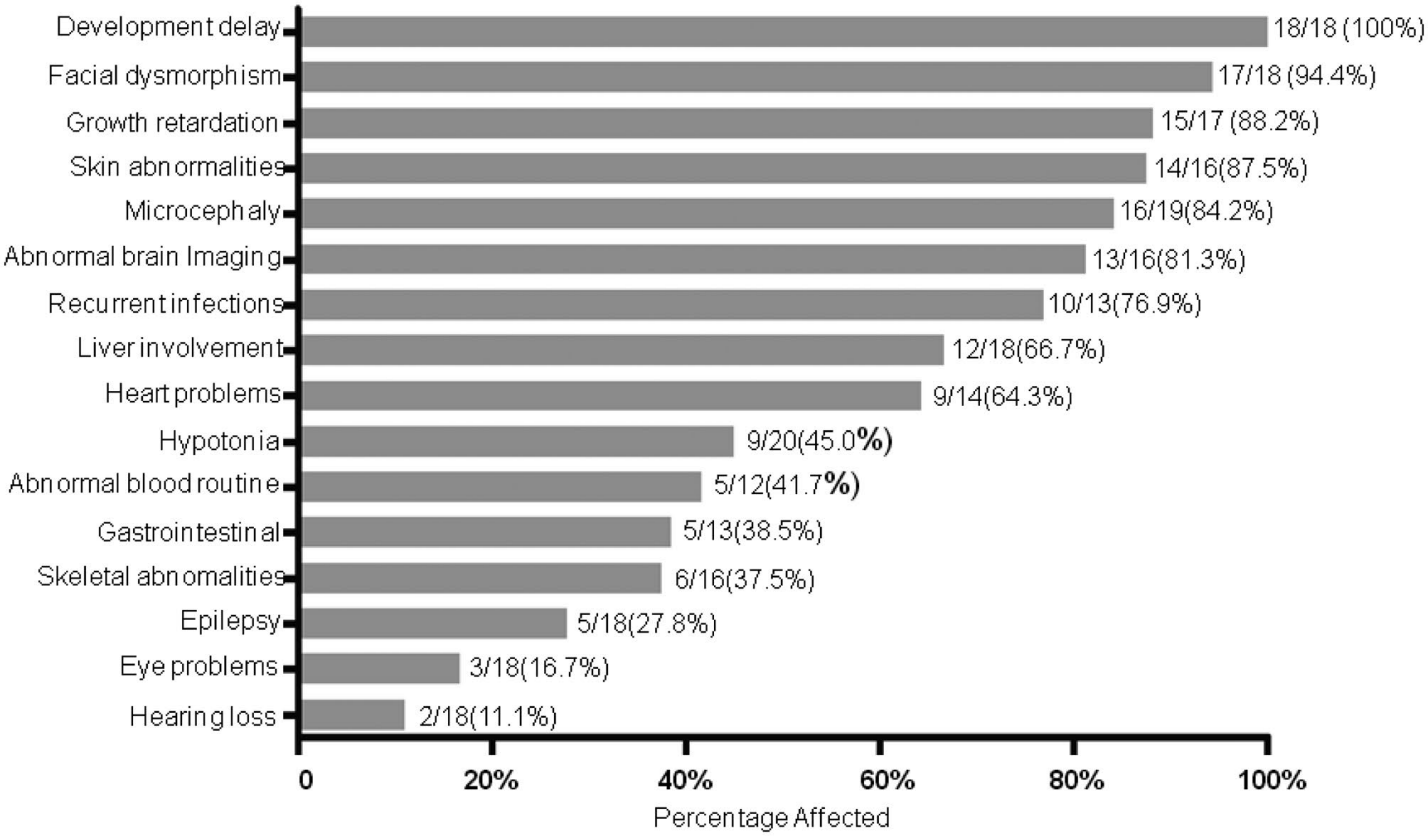
Phenotype summary from the 20 COG6‐CDG cases

We summarized the clinical manifestations of patients reported by reviewing the literatures and found that microcephaly is a consistent clinical hallmark of COG6‐CDG, which could be found at birth or in later development. It is proposed that microcephaly or other cortical dysplasia is likely due to reduced brain growth or damaged migration of neurons and formation of synapses during brain development (Kodera et al., 2015; Rymen et al., 2015). As shown in Figure 3, the most frequent features of COG6‐CGD patients are developmental disability (18/18) followed by facial dysmorphisms (17/18) and growth retardation (15/17). Skin abnormalities are also very common, including hyperkeratosis/hyperthermia, dry skin, orange peel skin, red rash, and hypohidrotic ectodermal dysplasia. Hypohidrosis may be caused by abnormal function of sweat glands as a normal structure and the density of eccrine sweat glands was observed in skin biopsy (Rymen & Jaeken, 2014). Recurrent infections and liver involvement are severe complications, which could be a critical cause of death (Rymen et al., 2015). Heart defects, including atrial septum defect, ventricular septal defect, and patent ductus arteriosus, are another feature and observed in more than half of COG6‐CDG patients (9/14). In addition, COG6‐CDG patients have some other symptoms, such as hypotonia, skeletal and gastrointestinal abnormalities, epilepsy, and vision and hearing problems. Compared to these patients with COG6‐CDG, our patient had the typical clinical manifestations, such as developmental delay, facial dysmorphism, growth retardation, hypotonia, hypohidrosis, microcephaly, abnormal brain imaging, liver dysfunction, recurrent infection, and gastrointestinal dysfunction. However, there is no obvious heart problem, vision defect, hearing loss, and epilepsy. Combined the genetic variants identified in this patient, the diagnosis of COG6‐CDG was made (Table 1).

**TABLE 1:** Clinical characteristics of patients with COG6‐CDG

Patients	Present study	P1 (Rymen, et al)	P2 (Rymen, et al)	P3 (Rymen, et al)	P4 (Rymen, et al)	P5 (Rymen, et al)	P6 (Rymen, et al)		P7 (Rymen, et al)	P8 (Lübbehusen et al)	P9 (Huybrechts, et al)
Sex	F	F	M	F	M	M		F	F	F	F
Age of onset	8 months	1 month	1 year	6 months	21 years	3 months		At birth	1 month	NA	3 months
Age of being reported	20 months	–	–	–	21 years	–		–	12 years	–	6 years
Age of death	–	1 month	12 months	15 months	–	14 months		5 weeks	–	5 weeks	–
Ethnicity	Chinese	Bulgarian	Turkish	Turkish	Moroccans	Moroccans		Moroccans	Turkish	Turkish	Moroccans
Facial dysmorphism	+	+	+	+	+	–		NA	+	NA	+
Developmental delay	+	+	+	+	+	+		NA	+	NA	Mild
Growth retardation	+	+	+	+	–	–		NA	+	NA	+
Microcephaly	+	+	+	+	+	+		+	+	NA	+
Hypotonia	–	+	+	–	–	–		+	–	–	–
Brain MRI	Lesions of bilateral temporal, occipital, and parietal lobes	Agenesis of corpus callosum	Asymmetric lateral ventricle and Cerebrospinal fluid increase	Atrophy of brain and cerebellum	NA	NA		–	Atrophy of cerebral cortex	NA	–
Congenital heart disease	ASD/PFO	ASD, PDA	NA	ASD, PDA	NA	NA		ASD	VSD	NA	NA
Hepatobiliary abnormalities	Cholestasis, abnormal liver function	HMG, Cholestasis	HMG	HMG	HMG	HMG, Cholestasis, Hepatic failure		HMG, Cholestasis	HMG, Cholestasis, Cirrhosis	Cholestasis	Cirrhosis
Gastrointestinal	–	NA	Chronic diarrhea	Chronic diarrhea	–	NA		Intestinal ischemia	Chronic diarrhea	NA	Enteritis
Recurrent infection	+	–	+	+	+	+		–	+	–	+
Skin abnormalities	–	NA	NA	Hyperkeratosis	Hyperkeratosis	Dry skin		NA	Orange peel skin	NA	NA
Blood routine examination	–	Thrombocytopenia	Anemia, thrombocytopenia	Thrombocytopenia, leukocytosis	Thrombocytopenia, pancytopenia	NA		NA	Mild pancytopenia	NA	NA
skeleton	NA	Joint contracture, adduction of thumb	–	Postaxial polydactyly	–	–		NA	Scoliosis	NA	Postaxial polydactyly
Seizure	Abnormal EEG	+	–	–	–	–		–	–	+	NA
Hearing/ophthalmological abnormality	–	Optic atrophy	Hearing loss	NA	Hearing loss	NA		–	–	–	–
Others	–	Hyperechoic kidney, hypotonia	NA	Immunodeficiency	Monocytosis	NA		Thymus hyperplasia, adrenal hypoplasia	Unilateral renal hypoplasia	intracranial hemorrhage	Combined immunodeficiency
Allele 1	c.1843C>T (p.Q615X)	c.511C>T (p.R171X)	c.1746+2T>G	c.1238_1239insA (p.F414Lfs*4)	c.1646G>T (p.G594V)	c.1646G>T (p.G594V)		NA*	c.511C>T (p.R171X)	c.1646G>T (p.G594V)	c.1646G>T (p.G594V)
Allele 2	c.428G>T (p.S143I)	c.511C>T (p.R171X)	c.1746+2T>G	c.1238_1239insA (p.F414Lfs*4)	c.785A>G (p.T262C)	c.785A>G (p.T262C)		NA	c.1746+2T>G	c.1646G>T (p.G594V)	c.1646G>T (p.G594V)

Patients	P10 (Shaheen, et al)	P11 (Li, et al)	P12 (Alsubhi, et al)	P13 (Alsubhi, et al)	P14 (Alsubhi, et al)	P15 (Alsubhi, et al)	P16 (Alsubhi, et al)	P17 (Alsubhi, et al)	P18 (Alsubhi, et al)	P19 (Wu, et al)
Sex	M	M	M	M	F	M	F	F	M	F
Age of onset	1 week	1 month	NA	NA	NA	NA	NA	NA	NA	2 months
Age of being reported	13 years	28 months	–	13 years	2 years	8 years	4 years	3 years	7 years	–
Age of death	–	–	3 months	–	–	–	–	–	–	Dead (unclear detail)
Ethnicity	Saudi	Chinese	Saudi	Saudi	Saudi	Saudi	Saudi	Saudi	Saudi	Chinese
Facial dysmorphism	+	+	+	+	+	+	+	+	+	+
Developmental delay	+	+	+	+	+	+	+	+	+	NA
Growth retardation	NA	+	+	+	+	+	+	+	+	+
Microcephaly	–	Mild	+	+	–	+	+	+	+	–
Hypotonia	–	–	+	+	+	–	+	+	+	–
Brain MRI	NA	abnormal signaling of the long T1 and long T2 in the right frontal region	Agenesis of corpus callosum	Hypo−myelination	PVL, reduced WM, thin splenium of corpus callosum	–	Delayed myelination, thin corpus callosum	Thin corpus callosum	Brain atrophy, PVL, bright T2 along corticospinal tract	Bilateral ventricles are slightly full
Congenital heart disease	NA	VSD/PFO	Dysplastic aortic valve, PDA	–	VSD/PFO	–	–	–	–	VSD/PFO
Hepatobiliary abnormalities	NA	–	–	–	–	–	–	–	NA	Abnormal liver function
Gastrointestinal	NA	–	NA	NA	NA	NA	NA	NA	NA	Gas accumulation
Recurrent infection	+	+	NA	NA	NA	NA	NA	NA	NA	+
Skin abnormalities	Hyperkeratosis	red rash	Lipodystrophy	HH	HH	HH	–	Lipodystrophy	HH	Hypohidrotic ectodermal dysplasia
Blood routine examination	NA	NA	NA	NA	NA	NA	NA	NA	NA	Anemia
Skeleton	NA	–	+	–	–	+	–	–	–	–
Seizure	NA	+	–	+	–	–	–	–	–	–
Hearing and eye problems	–	–	Strabismus	–	–	+	–	–	Bilateral ptosis	–
Others	NA	Elevation of myocardial enzymes	Cerebellar hypoplasia	Cerebellar hypoplasia	NA	NA	NA	Autism		Left kidney crystal
Allele 1	c.1167‐24A>G (p.G390FfsX6)	c.1A>G (p.M1V)	c.1378G>T (p.V460F)	c.1167‐24A>G (p.G390FfsX6)	c.1167‐24A>G (p.G390FfsX6)	c.1167‐24A>G (p.G390FfsX6)	c.1167‐24A>G (p.G390FfsX6)	c.1075‐9T>G	c.1167‐24A>G (p.G390FfsX6)	c.511C>T (p.R171X)
Allele 2	c.1167‐24A>G (p.G390FfsX6)	c.388C>T (p.Q130X)	c.1378G>T (p.V460F)	c.1167‐24A>G (p.G390FfsX6)	c.1167‐24A>G (p.G390FfsX6)	c.1167‐24A>G (p.G390FfsX6)	c.1167‐24A>G (p.G390FfsX6)	c.1075‐9T>G	c.1167‐24A>G (p.G390FfsX6)	c.540G>A (p.E180E)

HH, Hypohydrosis; HMG, hepatomegaly; NA, not available.

* P6 was the sister of P8, dead at the age of 5 weeks, and not done genetic test. GenBank reference sequence and version number: NG_028352.1.

As reported by Rymen and her colleagues, patients with splice site mutation had mild clinical phenotype especially in patients with c.1167‐24A>G mutation (Rymen et al., 2015). All six patients with homozygous c.1167‐24A>G mutation had only mild to moderate developmental delay and mental retardation and no abnormality in liver function, gastrointestinal system, and skeletal system (Alsubhi et al., 2017; Li et al., 2019; Shaheen et al., 2013). Only one of the six patients had heart problems, skeletal abnormalities, or recurrent infection, but all six patients presented skin abnormalities. However, both patients with homozygous loss‐of‐function mutations p.Arg171* or p.Phe414Leufs*4 die at the age of 1 month and 15 months due to respiratory or liver failure, respectively (Rymen et al., 2015). So, all findings suggest that patients’ harboring nonsense mutations may present more severe clinical manifestations. These results need to be further confirmed by studying more patients.

## 6 CONCLUSION

We identified two compound heterozygous variants in *COG6* gene in a patient with developmental delay, facial dysmorphism, microcephaly, recurrent infection, and hypohidrosis. A prenatal diagnosis was performed by amniocentesis for this family in the subsequent pregnancy. Our work broadens the mutation spectrum of *COG6* gene and also emphasizes the importance of whole‐exome sequencing in definitive diagnosis and prenatal diagnosis in the subsequent pregnancy.

## ETHICS APPROVAL AND CONSENT TO PARTICIPATE

The study was done after obtaining the ethics approval from the institutional review board of Wuhan Children's Hospital, Tongji Medical College, Huazhong University of Science and Technology (approval number 2019011), and written informed consent from the patient's parents.

## CONFLICT OF INTEREST

The authors declare that they have no competing interests.

## AUTHORS’ CONTRIBUTIONS

Study concepts: XH, PZ. Study design: PZ, PH, JC. Literature research: LZ. Clinical information collection: HP, JC. Data acquisition: PZ, SL. Data analysis/interpretation: LT, YH. Manuscript preparation: PZ, PH. Manuscript editing/revision/review: XH, JC. All authors have read and approved the manuscript.

## Data Availability

The data and materials are submitted and are available on reasonable request.
